# Localized heterochrony integrates overgrowth potential of oncogenic clones

**DOI:** 10.1242/dmm.049793

**Published:** 2023-02-08

**Authors:** Nicola Blum, Matthew P. Harris

**Affiliations:** ^1^ Department of Orthopaedics, Boston Children's Hospital, 300 Longwood Avenue, Boston, MA 02115, USA; ^2^ Department of Genetics, Harvard Medical School, 77 Avenue Louis Pasteur, Boston, MA 02115, USA

**Keywords:** Zebrafish, Skeleton, Somatic mutation, Macrodactyly, Proteus syndrome, Cartilage condensation

## Abstract

Somatic mutations occur frequently and can arise during embryogenesis, resulting in the formation of a patchwork of mutant clones. Such mosaicism has been implicated in a broad range of developmental anomalies; however, their etiology is poorly understood. Patients carrying a common somatic oncogenic mutation in either *PIK3CA* or *AKT1* can present with disproportionally large digits or limbs. How mutant clones, carrying an oncogenic mutation that often drives unchecked proliferation, can lead to controlled and coordinated overgrowth is unknown. We use zebrafish to explore the growth dynamics of oncogenic clones during development. Here, in a subset of clones, we observed a local increase in proportion of the fin skeleton closely resembling overgrowth phenotypes in patients. We unravel the cellular and developmental mechanisms of these overgrowths, and pinpoint the cell type and timing of clonal expansion. Coordinated overgrowth is associated with rapid clone expansion during early pre-chondrogenic phase of bone development, inducing a heterochronic shift that drives the change in bone size. Our study details how development integrates and translates growth potential of oncogenic clones, thereby shaping the phenotypic consequences of somatic mutations.

## Teaser

**Summary:** Overproliferation of pre-chondrogenic cells carrying a somatic oncogenic mutation in *AKT1* or *PIK3CA* cause a heterochronic shift in long bone formation and skeletal gigantism.

## INTRODUCTION

With the advent of whole-genome sequencing of somatic tissues and cellular bar coding, it is becoming clear that somatic mutations are quite common (Biesecker and Spinner, 2013; García-Nieto et al., 2019; Li et al., 2021). Starting in the early embryo, somatic mutations accumulate in our cells throughout life, leading to a patchwork of mutant cell clones in our tissues and organs. Among mutant clones, a small minority may harbor a mutation that increases the growth potential of a cell. Such clones have long been known to cause cancer, and it is becoming increasingly evident that a broad range of developmental disorders are also products of somatic oncogenic mutations (Moog et al., 2020; Mustjoki and Young, 2021; Nussinov et al., 2022).

PIK3CA-related overgrowth spectrum (PROS) and Proteus syndrome are extremely rare conditions characterized by localized overgrowths that can affect virtually any tissue and organ (Doucet et al., 2016; Keppler-Noreuil et al., 2014, 2015; Kurek et al., 2012; Lindhurst et al., 2011, 2019; Venot et al., 2018). Somatic activating mutations of phosphatidylinositol 4,5-bisphosphate 3-kinase catalytic subunit alpha isoform (*PIK3CA*) and AKT serine/threonine kinase 1 (*AKT1*) have been detected in patients with PROS and Proteus syndromes, respectively (*PIK3CA*^H1047R^, *PIK3CA*^E542K^, *PIK3CA*^E545K^, *PIK3CA*^G1049R^ and *AKT1^E17K^*). These mutations are well-known oncogenic mutations (Chen et al., 2018; Karakas et al., 2006; Lawrence et al., 2014; Rudolph et al., 2016; Shoji et al., 2009) that render the phosphoinositide 3 kinase (PI3K)/AKT pathway – a key regulator of cell growth, proliferation and survival – hyperactive (Hoxhaj and Manning, 2020). In both syndromes, the particular mutation does not, however, define the particular presentation as the same mutations have been found to drive a suite of different disorders. PROS and Proteus syndrome are remarkably heterogeneous disorders. Thus, phenotypes arising from these somatic mutations are likely the result of modifying genetic or environmental factors, or, alternatively, the timing and location of the mutation event in the embryo influencing phenotypic manifestation. The underlying mechanisms detailing the particular phenotypic expressivity of clones with oncogenic PI3K/AKT signaling have not been determined.

**Fig. 1.**
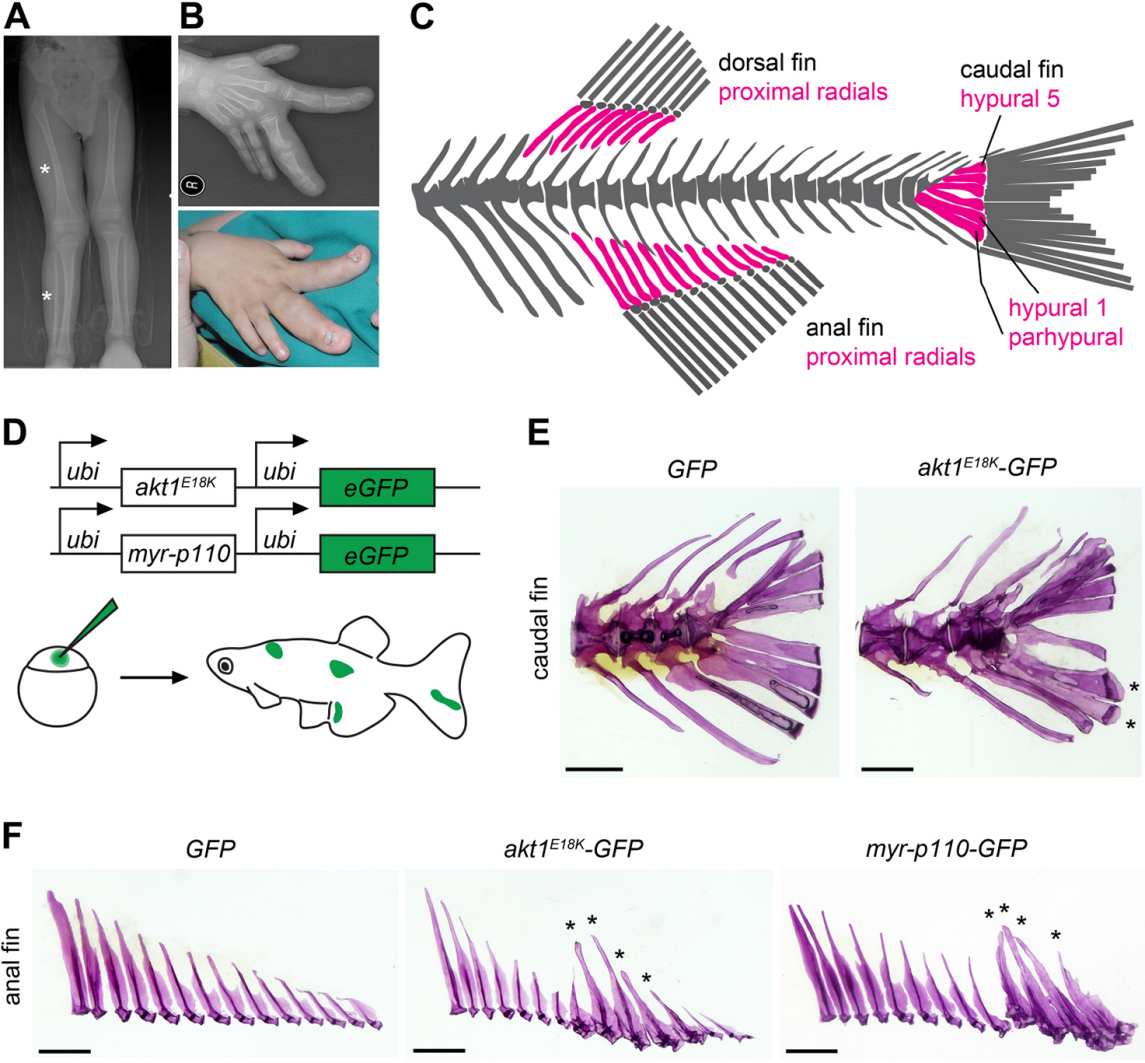
**Mosaic overactivation of the PI3K/AKT pathway causes fin long bone overgrowth in zebrafish.** (A) Leg length discrepancy due to overgrowth of the right tibia and fibula (marked by asterisks) in a patient with Proteus syndrome. (B) Macrodactyly in a patient diagnosed with PROS. Images in A and B were modified from Zeng et al. (2020) and Tian et al. (2020), respectively, under the terms of the original CC-BY 4.0 license. (C) Generalized diagram of the adult zebrafish axial skeleton. Long bones of the median fin endoskeleton are highlighted in magenta. (D) Schematic of the experimental design to generate mosaic zebrafish harboring clones with oncogenic PI3K/AKT signaling. (E,F) Representative stereomicroscope images of fin long bone overgrowth observed in *akt1^E18K^-GFP* and *myr-p110-GFP* mosaic fish. Alizarin Red-stained preparations of caudal (E) and anal (F) fin endoskeleton. Asterisks mark overgrown bones. Scale bars: 0.1 cm.

In patients diagnosed with PROS or Proteus syndrome, overgrowth is often uncoordinated, disrupting normal tissue patterning and relative proportions. There are interesting exceptions, where patients present with localized gigantism in the appendicular skeleton (Fig. 1A,B) (Bornstein et al., 2014; Cerrato et al., 2013; Keppler-Noreuil et al., 2014, 2015; Rios et al., 2013; Tian et al., 2020; Tripolszki et al., 2016; Wu et al., 2018; Zeng et al., 2020). In such patients, affected long bones are longer and wider, overall retaining their normal shape, indicating that growth is controlled and coordinated. The result is an abnormally large limb or digit (macrodactyly). In the latter case, more than one, but always adjacent, digits are often affected, suggesting regional affected territories. How somatic oncogenic mutations in the PI3K/AKT pathway associated with unchecked proliferation and a broad range of neoplasms can lead to controlled and coordinated overgrowth of entire entities provides a useful case study to investigate how growth potential is shaped during development. Here, we develop a zebrafish model for overgrowth phenotypes as observed in PROS and Proteus syndrome, and explore the underlying mechanisms of long bone overgrowth. Our findings revealed that overgrowth of mutant clones is restricted to the pre-chondrogenic phase during bone development. Interestingly, the rapid expansion of mutant clones during this early stage caused premature skeletal condensation and this shift in developmental timing drives the observed non-neoplastic overgrowth of entire long bones.

## RESULTS

### Mosaic overactivation of PI3K/AKT signaling causes localized gigantism of the fin endoskeleton

To understand how mutant clones that harbor an oncogenic mutation in either PIK3CA or AKT1 can drive controlled overgrowth of long bones, we thought to employ a model that allows *in vivo* imaging of mutant cell clones. To this end, we made use of zebrafish as we can readily make clones and observe them in real time during development and into adulthood (e.g. Perathoner et al., 2014). The endoskeleton of zebrafish median fins (caudal, anal and dorsal fin) harbors a series of endochondral bones (Fig. 1C), called hypurals (hypural 1-5) and parhypural in the caudal fin and proximal radials in dorsal and anal fins (Bird and Mabee, 2003). Of note, fin long bones have characteristic size ratios; for example, in the anal fin, bone length decreases from anterior to posterior. Thus, overgrowth of individual bones can be readily visible. The cellular and molecular mechanisms underlying development, patterning and growth of long bones in the teleost fin are very similar to those in the tetrapod limb (Crotwell and Mabee, 2007; Hawkins et al., 2021). Thus, the zebrafish provides a functional model to investigate effects of somatic mutations in the appendicular skeleton.

In Proteus syndrome and PROS, the origin of the causative mutation is believed to occur during early development, thus resulting in mutant cell clones scattered throughout the body and across many cell types. It is unknown which cell type drives overgrowth and harbors the mutation in affected long bones. We therefore aimed to generate mosaic zebrafish harboring cell clones with overactivated PI3K/AKT signaling randomly distributed across all tissues. To this end, we used a Tol2 transposase to allow random, mosaic, stable transgene integrations during early embryonic development (Fig. 1D) (Kawakami, 2007). Transgenic constructs harbor either *akt1^E18K^* – i.e. the corresponding mutation of human *AKT^E17K^* in zebrafish – or a constitutively active form of murine *PIK3CA* (*myr-p110α*) under the control of the ubiquitously active *ubiquitin* (*ubi*) promoter. Both constructs express the fluorescent marker enhanced green fluorescent protein (eGFP) under the control of a second *ubi* promoter to track the presence of clones. Hereafter, we refer to the transgenic constructs as *akt1^E18K^-GFP*, *myr-p110-GFP* and *GFP* (control).

We found that high numbers of clones expressing *akt1^E18K^-GFP* or *myr-p110-GFP* lead to early lethality at larval stages due to severe vascular abnormalities and hemorrhaging. This is in congruence with previous findings in PROS mouse models (Venot et al., 2018). Vascular abnormalities are also common in patients suffering from PROS or Proteus syndrome (Castillo et al., 2016; Detter et al., 2018; Luks et al., 2015; Perry et al., 2007; Queisser et al., 2018; Rodriguez–Laguna et al., 2019). To accommodate the observation of clones with overactivated PI3K/AKT signaling during later developmental stages as well as in adults, we decreased the number of transgene-expressing clones to minimize lethality. Even under these experimental conditions, ∼50% of injected fish harbored clones in multiple tissues (Fig. S1A).

mTor signaling is a primary mediator of PI3K/AKT signaling (Hoxhaj and Manning, 2020). By immunostaining of injected larvae at 7 days post fertilization [3.7 mm standard length (SL)] with an anti-phospho-S6 ribosomal protein (pS6) antibody, a commonly used readout of mTor pathway activation, we assessed the activity of the PI3K/AKT pathway. We detected higher pS6 levels in *akt1^E18K^-GFP-* and *myr-p110-GFP-*expressing cells compared to control cells expressing only *GFP* (Fig. S1B; 15 analyzed fish per transgene), indicating enhanced PI3K/AKT signaling.

To verify that *akt1^E18K^-GFP* and *myr-p110-GFP* mosaic animals mimic overgrowth phenotypes as seen in patients diagnosed with PROS or Proteus syndrome, we analyzed adult fish (3-6 months old) for externally visible overgrowth phenotypes. Indeed, *akt1^E18K^-GFP* and *myr-p110-GFP* mosaic fish showed localized or patchy overgrowth in the skin, adipose tissue, muscle and vasculature (Fig. S2). We next analyzed the endoskeleton of median fins in adult mosaic fish by Alizarin Red staining. Owing to the random distribution of clones, only a small percentage of injected fish is expected to harbor the transgene in the correct cell type and location to cause long bone overgrowth. We found disproportionally large fin long bones in several *akt1^E18K^-GFP* and *myr-p110-GFP* mosaic fish (*akt1^E18K^-GFP*: caudal fin *n*=7, anal fin *n*=13, dorsal fin *n*=11, analyzed fish *n*=203; *myr-p110-GFP*: caudal fin *n*=5, anal fin *n*=8, dorsal fin *n*=5, analyzed fish *n*=132) compared to control-injected fish (GFP: caudal fin *n*=0, anal fin *n*=0, dorsal fin *n*=0, analyzed fish *n*=250) (Fig. 1E,F). Affected bones were longer and wider yet, generally, showed normal shape. In most cases, two or more bones, which were always adjacent, were affected. These structures closely resemble long bone overgrowth observed in patients diagnosed with PROS or Proteus syndrome, revealing an actuatable model for this disorder in zebrafish. No apparent differences in severity or frequency were found between different median fins as well as between *akt1^E18K^-GFP* and *myr-p110-GFP* mosaic fish, indicating that the two transgenes and different median fins can be used interchangeably to model this phenotype.

### Long bone overgrowth is caused by a heterochronic shift in development of the cartilage anlagen

**Fig. 2.**
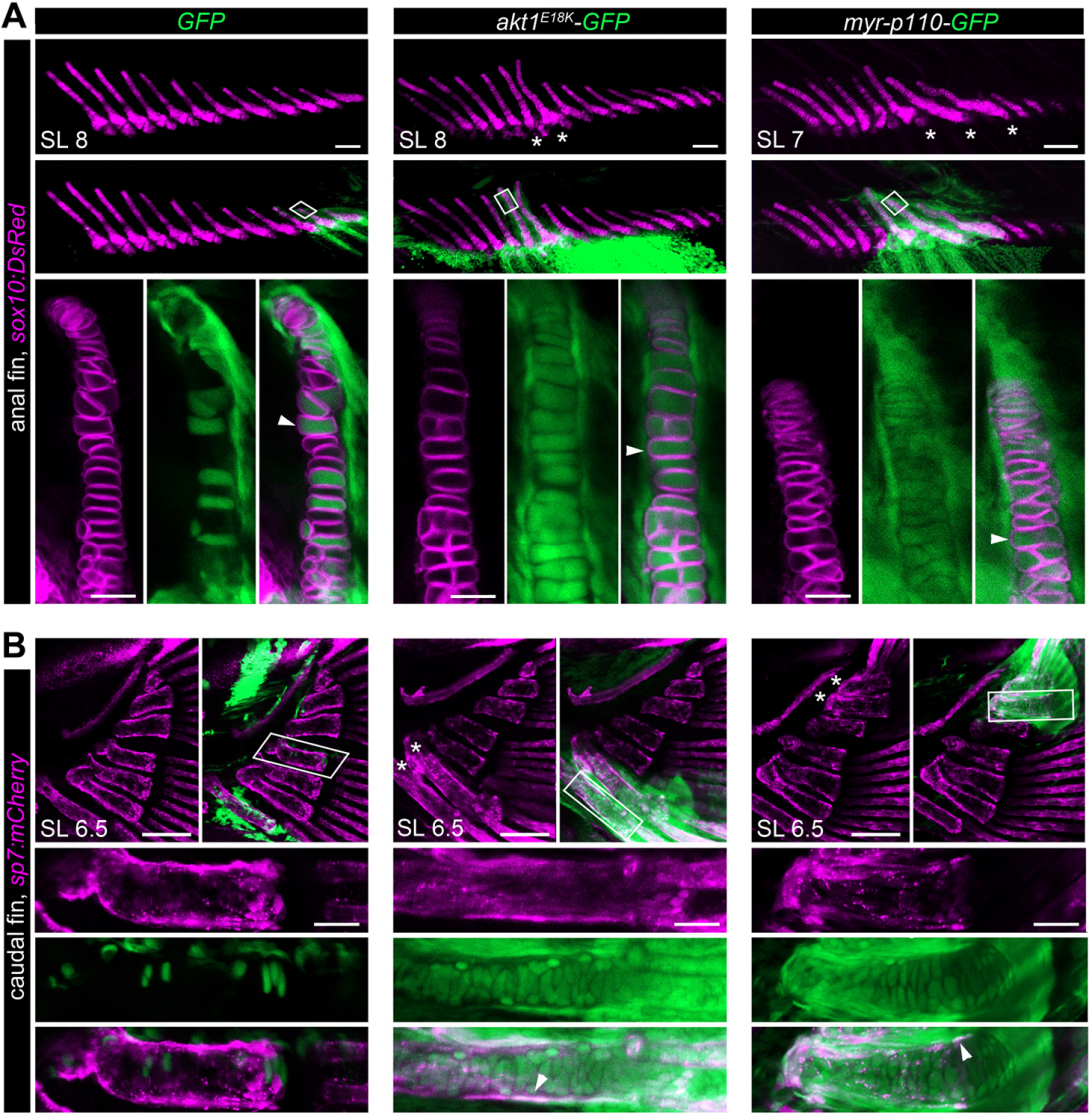
**Long bone overgrowth is caused by PI3K/AKT overactivation in mesenchymal derivatives.** PI3K/AKT mosaic overactivation results in an early overgrowth phenotype in the fin endoskeleton. (A,B) *In vivo* confocal imaging of chondrocytes (A) marked by *Tg(sox10:DsRed)* (magenta) and osteoblasts (B) marked by *Tg(sp7:mCherry)* (magenta) in late larval stages reveals localized skeletal overgrowth in median fins of *akt1^E18K^-GFP* (green) and *myr-p110-GFP* (green) mosaic larva overlapping with GFP^+^ cells. Boxed areas are at high magnification, showing that *akt1^E18K^-GFP* and *myr-p110-GFP* colocalize with chondrocytes and osteoblasts. Asterisks indicate overgrown bones. Arrowheads indicate GFP^+^ chondrocytes in A and GFP^+^ osteoblasts in B. SL, standard length in mm. Scale bars: 100 μm; high-magnification images: 25 μm.

To determine which cell type drives the observed long bone overgrowth, we generated *akt1^E18K^-GFP* and *myr-p110-GFP* clones in transgenic zebrafish reporter lines for either chondrocytes [*Tg(sox10:DsRed)*] (Das and Crump, 2012) or osteoblasts [*Tg(Ola.Sp7:mCherry)*] (DeLaurier et al., 2010) to visualize the fin endoskeleton in live larval and juvenile fish. *In vivo* imaging at 6.5-9 mm SL revealed that long bone overgrowth was already present at late larval stages (*akt1^E18K^-GFP*: caudal fin *n*=50, anal fin *n*=63, dorsal fin *n*=72, analyzed fish *n*=1225; *myr-p110-GFP*: caudal fin *n*=39, anal fin *n*=42, dorsal fin *n*=38, analyzed fish *n*=1052) (Fig. 2A,B). However, no changes were observed in control fish (GFP: caudal fin *n*=0, anal fin *n*=0, dorsal fin *n*=0, analyzed fish *n*=920). In all overgrown skeletal elements observed, *akt1^E18K^-GFP* and *myr-p110-GFP* colocalized with chondrocyte and osteoblast markers. We also detected transgene expression in cells that surrounded overgrown bones. Based on cell morphology and location, these cells were likely to be fibroblasts. Thus, the change in the relative size of long bones observed is caused by PI3K/AKT overactivation in the mesenchymal lineage.

**Fig. 3.**
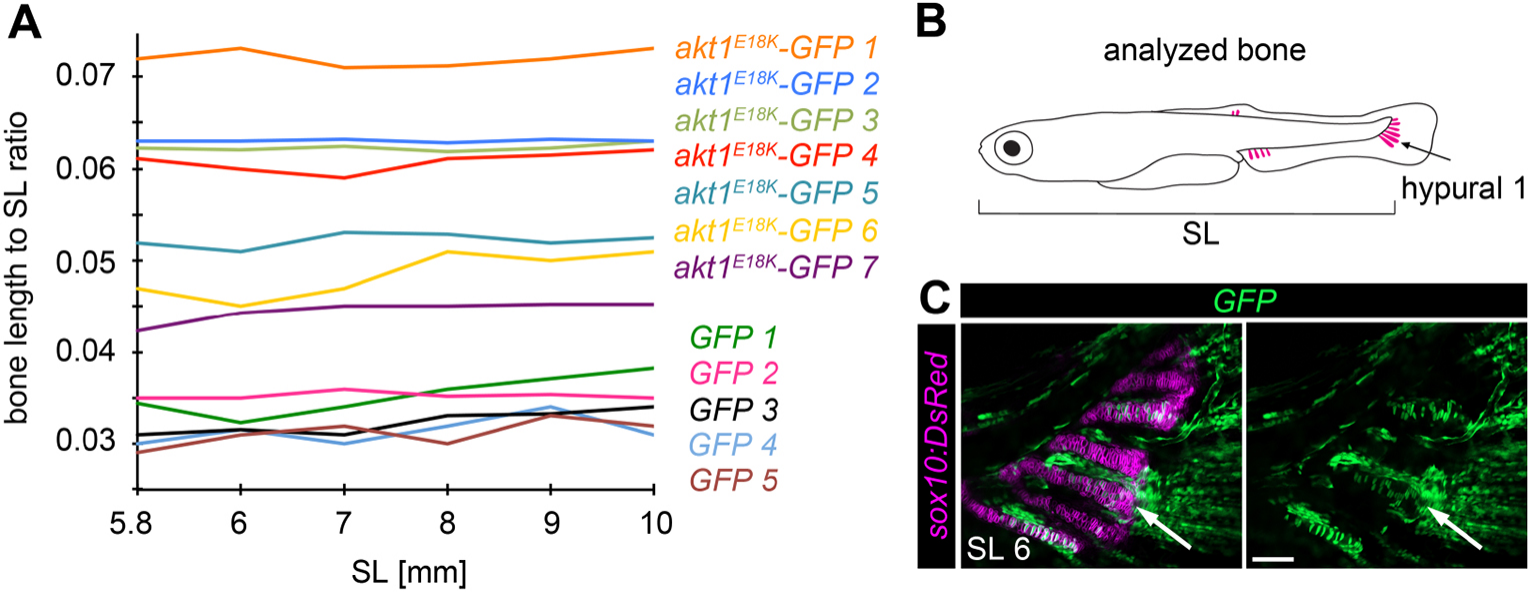
**Long bone overgrowth is non-progressive.** Overgrown long bones in *akt1^E18K^-GFP* mosaic larvae elongate at the same rate as bones in control larvae. (A) Ratio of the length of endochondral bone hypural 1 to SL over time (SL 5.8-10 mm) in *akt1^E18K^-GFP* (*n*=7) and *GFP* (*n*=5) mosaic larvae. (B) Analyzed bone (GFP^+^ hypural 1 of the caudal fin). (C) Representative *in vivo* confocal image of the caudal fin of an analyzed *GFP* (green) mosaic larva. *Tg(sox10:DsRed)* marks chondrocytes (magenta). Arrows mark hypural 1. SL, standard length in mm. Scale bar: 50 μm.

We next explored the developmental mechanisms by which *akt1^E18K^* and *myr-p110* modified bone size. There are three different options to achieve a change in relative bone size: (1) change in elongation rate, (2) change in developmental timing (heterochrony) or, (3) change in the size of cartilage anlagen. Similar to that of tetrapod long bones, elongation of long bones in fins is driven by proliferation of chondrocytes and subsequent hypertrophy at the end of bones. The PI3K/AKT pathway is a key regulator in promoting cell growth and proliferation, suggesting that *akt1^E18K^-GFP*- and *myr-p110-GFP*-positive chondrocytes possess increased growth potential. We, therefore, assumed that the observed change in bone size is due to accelerated bone elongation. Indeed, we found that, in contrast to control fish, all chondrocytes in affected long bones in *akt1^E18K^-GFP* and *myr-p110-GFP* mosaic fish harbored the transgene (Fig. 2A). This is indicative of a growth advantage that mutant cells have over wild-type cells. We then compared the rate of bone elongation in clone-harboring hypural 1 between *GFP* and *akt1^E18K^-GFP* mosaic fish from SL 5.8 mm to SL 10 mm. We found that overgrowth is not progressive, and affected bones were observed to grow at the same rate as bones in control larvae (Fig. 3). Thus, the observed change in relative long bone size is not caused by an increase in growth rate. Moreover, the fact that overgrowth of hypural 1 in *akt1^E18K^-GFP* mosaic fish was already present at SL 5.8 mm suggests that the growth advantage of PI3K/AKT mutant clones is restricted to early stages of bone development.

**Fig. 4.**
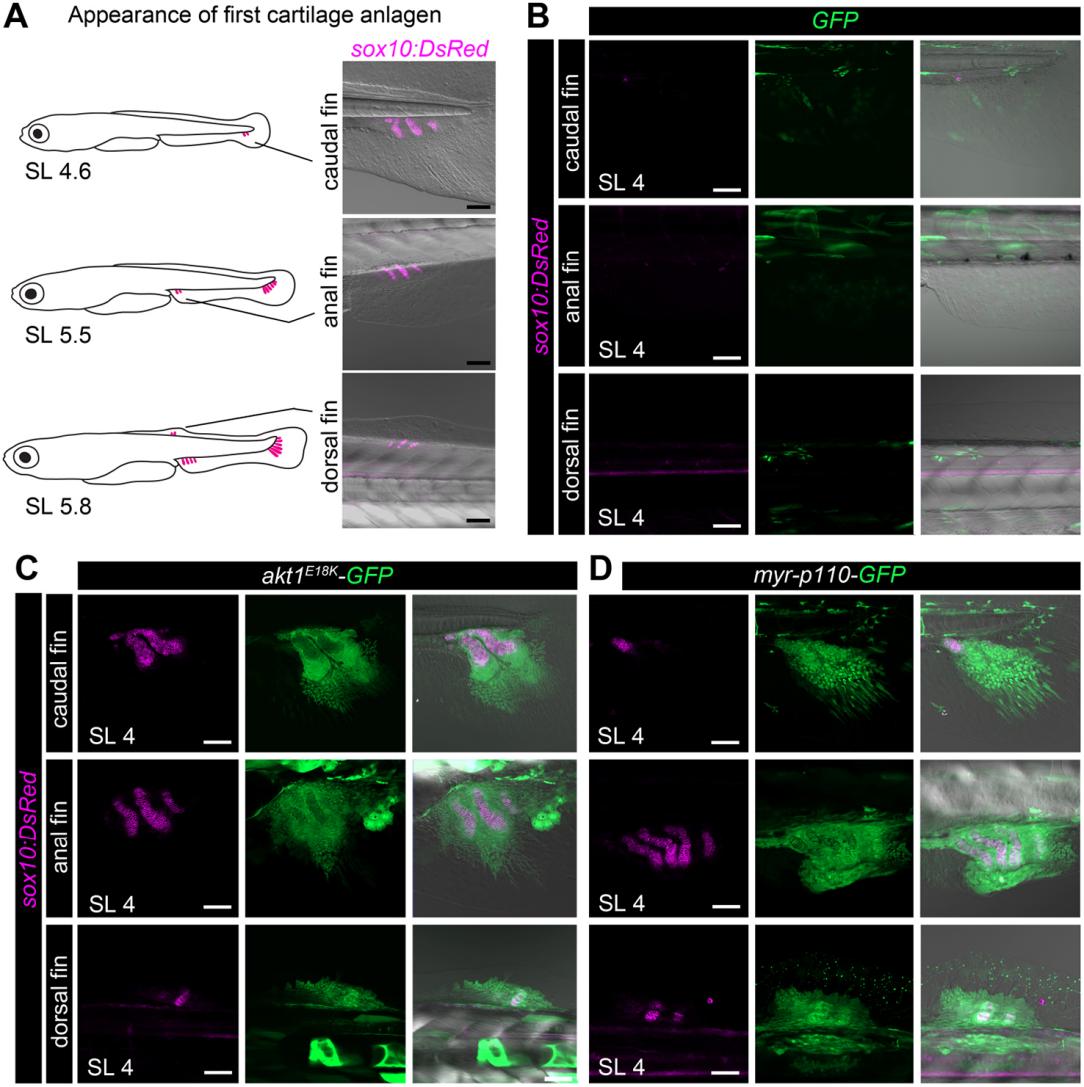
**PI3K/AKT mutant clones induce a heterochronic shift in long bone development.** (A) Schematic representation and *in vivo* confocal imaging of first cartilage anlagen [marked by *Tg(sox10:DsRed)* (magenta)] in caudal, anal and dorsal fins. (B-D) *In vivo* confocal imaging of chondrocytes [*Tg(sox10:DsRed)* (magenta)] at SL 4 mm in *GFP* (green) (B), *akt1*^*E18K*^*-GFP* (green) (C) and *myr-p110-GFP* (green) (D) mosaic larvae reveals premature formation of cartilage anlagen in median fins of *akt1*^*E18K*^*-GFP* and *myr-p110-GFP* larvae. SL, standard length in mm. Scale bars: 50 μm.

Next, we analyzed whether bone overgrowth is caused by a change in relative developmental timing. Using non-injected *Tg(sox10:DsRed)* fish, we found that the first cartilage anlagen appear at approximately SL 4.5 mm in the caudal fin (*n*=17), at SL 5.5 mm in the anal fin (*n*=15) and at SL 5.8 mm in the dorsal fin (*n*=19) (Fig. 4A). Surprisingly, in *akt1^E18K^-GFP* and *myr-p110-GFP* mosaic fish, cartilage anlagen with overlapping mesenchymal clones appeared prematurely as early as SL 4.0 mm in median fins (*akt1^E18K^-GFP*: caudal fin *n*=93, anal fin *n*=85, dorsal fin *n*=87, analyzed fish *n*=1500; *myr-p110-GFP:* caudal fin *n*=45, anal fin *n*=51, dorsal fin *n*=39, analyzed fish *n*=1032) (Fig. 4B-D). This was never observed in control fish (GFP: caudal fin *n*=0, anal fin *n*=0, dorsal fin *n*=0, analyzed fish *n*=1002). Taken together, these findings demonstrate that mesenchymal clones with overactivated PI3K/AKT signaling cause a heterochronic shift in formation of cartilage anlagen, and that this temporal modulation is the primary cause for the change in relative long bone size.

**Fig. 5.**
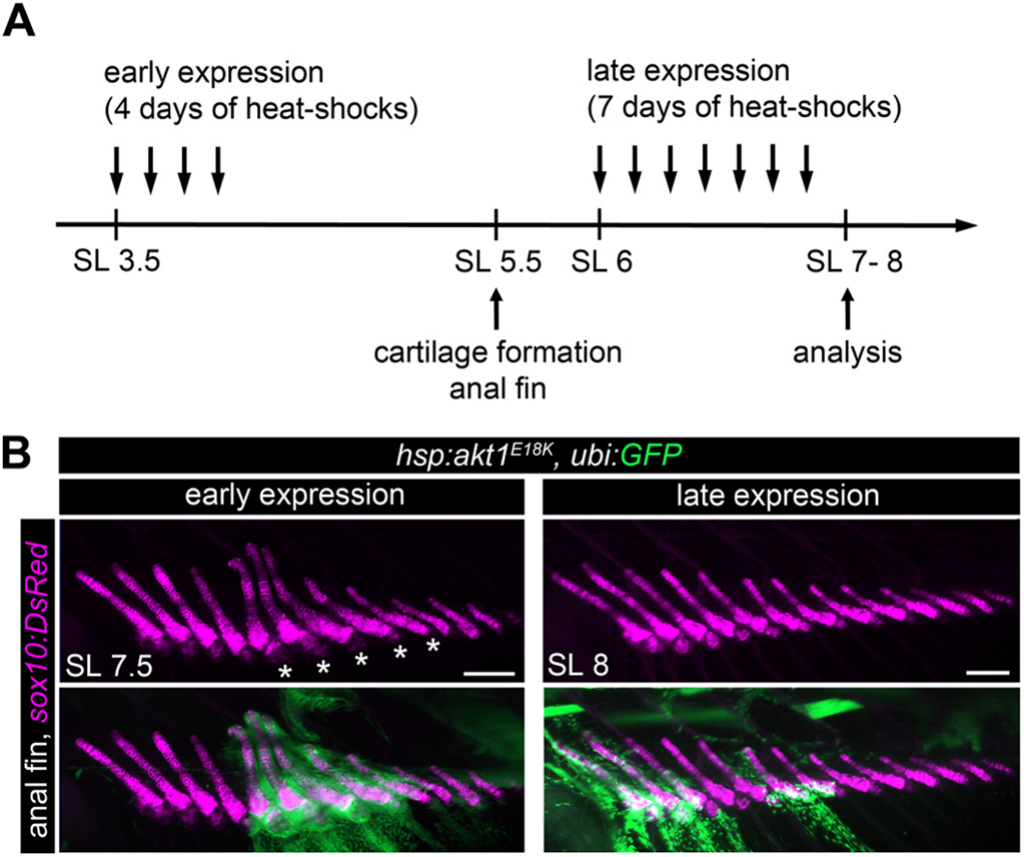
***akt1^E18K^* mosaic expression restricted to early larval stages is sufficient to cause long bone overgrowth.** (A) Schematic of the experimental design to analyze the temporal requirement of PI3K/AKT overactivation in long bone overgrowth. (B) Representative *in vivo* confocal images of the anal fin endoskeleton [chondrocytes are marked by *Tg(sox10:DsRed)* (magenta)] in *hsp:akt*^*E18K*^*, ubi:GFP* (green) mosaic larva. Heat-shock induced *akt1*^*E18K*^ expression prior to cartilage formation (early expression) caused overgrowth in clone-harboring long bones. In contrast, bone size was unaffected if *akt1*^*E18K*^ was expressed post cartilage formation (late expression). Asterisks mark overgrown bones. SL, standard length in mm. Scale bars: 100 μm.

To further support this conclusion, we generated a construct to temporally induce *akt1^E18K^* mosaic expression (*hsp70:akt1^E18K^, ubi:GFP*) using the heat-sensitive zebrafish *hsp70* promoter (Halloran et al., 2000). If premature cartilage formation is the main driver for long bone overgrowth in PI3K/AKT mosaic fish, then activation of *akt1^E18K^* expression before cartilage normally forms should be sufficient to cause a change in relative bone size. Indeed, we found that 4 days of heat-shock treatment starting at SL 3.5 mm resulted in overgrowth of clone-harboring long bones (caudal fin *n*=4, anal fin=5, dorsal fin *n*=7, analyzed fish *n*=195) (Fig. 5). We neither found an effect on long bone size in non-heat-shocked *hsp:akt1^E18K^, ubi:GFP* mosaic fish (analyzed fish *n*=251) nor in heat-shocked *GFP* (analyzed fish *n*=260) control mosaic fish. Expression of *akt1^E18K^* after cartilage anlagen had formed (7 days of heat-shock treatment starting at SL 6 mm), failed to cause an overgrowth phenotype (analyzed fish *n*=210) (Fig. 5).

### PI3K/AKT mutant clones form premature pre-cartilage condensations

To explore how PI3K/AKT mutant mesenchymal clones cause the observed heterochronic shift in cartilage formation, we examined the behavior of mutant clones prior to the appearance of cartilage anlagen in median fin development. Formation of cartilage anlagen is initiated as undifferentiated and seemingly homogenous pre-chondrogenic mesenchymal cells become closely juxtaposed forming a pre-chondrogenic condensation. Subsequently, these condensations initiate matrix deposition and differentiate into chondrocytes (Hall and Miyake, 1996). During the condensation process, critical cell-cell and cell-matrix interactions occur that are necessary to trigger chondrogenic differentiation. In zebrafish, pre-chondrogenic condensations of median fins are visible as thickenings in the fin fold and growth proceeds in a proximal to distal direction (Parichy et al., 2009).

**Fig. 6.**
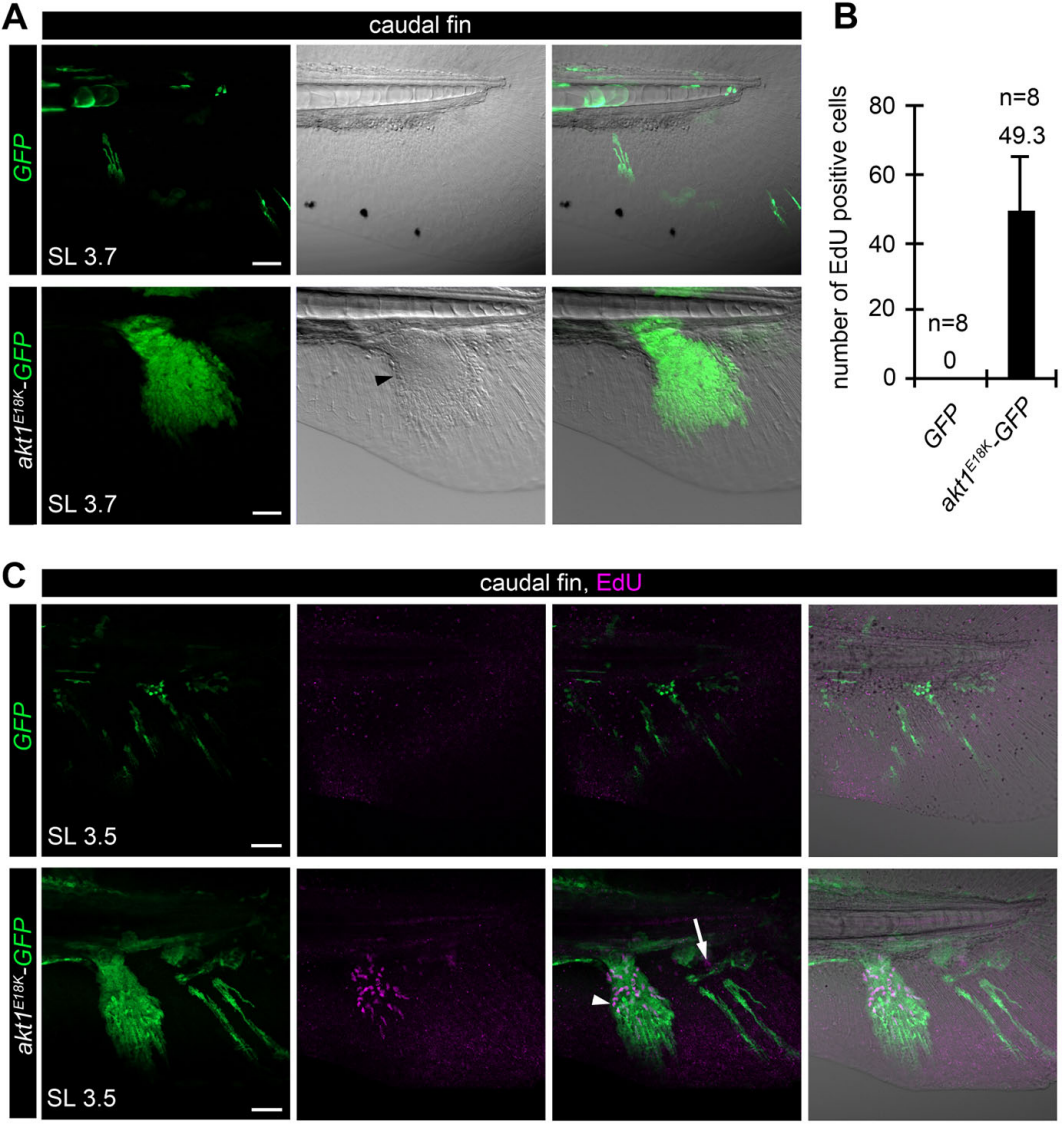
***akt1*^*E18K*^ mutant mesenchymal clones form premature pre-chondrogenic condensations.** (A) *In vivo* confocal imaging of *akt1^E18K^-GFP* (green) mesenchymal clones at SL 3.7 mm reveals clonal expansion and premature pre-chondrogenic condensation in the caudal fin. Arrowhead marks the edge of condensation. (B,C) *akt1^E18K^-GFP* mesenchymal clones in the larval fin are hyperproliferative. (B) Quantification of mesenchymal cells expressing GFP or *akt1^E18K^-GFP* labeled with 5-ethynyl-2′-deoxyuridine (EdU) to assay DNA synthesis in the caudal fin. Data are represented as mean±s.e.m. *n*=number of analyzed fish. (C) Representative *in vivo* confocal images of EdU (magenta) labeling in the caudal fin of *GFP* (green) and *akt1*^*E18K*^*-GFP* (green) mosaic larvae at SL 3.5 mm. Arrowhead marks *akt1*^*E18K*^*-GFP*-expressing EdU-positive cells. Arrow marks EdU-positive wild type cells. SL, standard length in mm. Scale bars: 50 μm.

We found that, during these early stages of fin development, *akt1^E18K^-GFP* and *myr-p110-GFP* mesenchymal clones were much larger than control clones (Fig. 6A; Fig. S3), suggestive of overproliferation of mutant cells. To confirm that increased clone size is, indeed, caused by overproliferation, we performed 5-ethynyl-2′-deoxyuridine (EdU) labeling to assay DNA synthesis in SL 3.5 mm *akt1^E18K^-GFP* mosaic larvae and analyzed the caudal fin. A short pulse of EdU did not result in labeling of fin mesenchymal cells in control fish (Fig. 6B,C), indicative for a slow proliferation rate at this stage. In contrast, we found a large number of EdU-labeled fin mesenchymal cells in *akt1^E18K^-GFP* mosaic fish (Fig. 6B,C). The majority (93%) of EdU-positive (EdU^+^) cells harbored the transgene. This result demonstrates that *akt1^E18K^-GFP* clones in the larval fin mesenchyme are hyperproliferative. We found a small number (7%) of EdU^+^ wild-type cells in proximity to mutant cells, suggesting that oncogenic AKT can exert non-cell-autonomous effects.

Intriguingly, *akt1^E18K^-GFP* and *myr-p110-GFP* mutant clones formed premature pre-chondrogenic condensations (*akt1^E18K^-GFP*: caudal fin *n*=101, anal fin *n*=90, dorsal fin *n*=93, analyzed fish *n*=1485; *myr-p110-GFP*: caudal fin *n*=45, anal fin *n*=34, dorsal fin *n*=35, analyzed fish *n*=1026) compared to controls (GFP: caudal fin *n*=0, anal fin *n*=0, dorsal fin *n*=0, analyzed fish *n*=1250) (Fig. 6A; Fig. S3). We conclude that mesenchymal clones with overactivated PI3K/AKT signaling cause a heterochronic shift in bone formation through premature pre-chondrogenic condensation. This shift occurs during early bone development, whereas later growth during bone elongation is not affected, resulting in proportional or coordinated changes in size.

## DISCUSSION

**Fig. 7.**
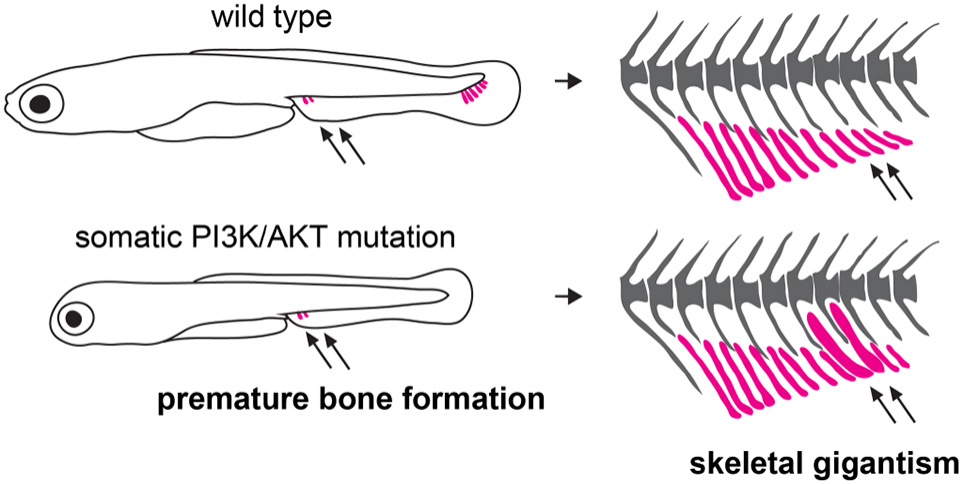
**Model for long bone overgrowth in PROS and Proteus syndrome.** Developmental mechanism underlying localized skeletal gigantism caused by somatic oncogenic mutations in the PI3K/AKT pathway. Long bones of the anal fin endoskeleton are highlighted in magenta.

Somatic mutations are increasingly found to be the underlying cause of many developmental disorders. However, we know very little how developmental context affects the behavior and phenotypic outcome of mutant clones, and whether these attributes can be leveraged to design interventions or treatments. By using a zebrafish model, we unraveled how overproliferation of oncogenic clones can lead to non-neoplastic and coordinated overgrowth of entire bones. We show that, during the earliest phase of bone development, the growth potential of oncogenic clones is translated into a heterochronic shift in cartilage formation, leading to localized and coordinated gigantism of the appendicular skeleton. Our findings support a model in which pre-chondrogenic cells that carry a somatic oncogenic mutation in *PIK3CA* or *AKT1* initiate cartilage development prematurely, thereby driving a qualitative change in the relative size of long bones (Fig. 7). Importantly, overgrowth in this context is not neoplastic, but coordinated, demonstrating a capacity within the developmental system for growth integration in these overgrowth conditions.

Pre-chondrogenic condensation is dependent on cell number (Osdoby and Caplan, 1979, 1980; San Antonio and Tuan, 1986). The burst in proliferation of PI3K/AKT mutant cartilage progenitors likely accelerates an increase in cell density prior to normal condensation. As a result, the critical threshold in cell number will be reached earlier. However, although high density of pre-chondrogenic cells is a requirement, this state is probably insufficient to induce cartilage template formation. Interestingly, mTor signaling –the primary mediator of PI3K/AKT signaling – has previously been shown to be required for pre-chondrogenic condensation through translational control of SOX9 (Iezaki et al., 2018). Our data showed pS6 immunoreactivity, which marks localized increase of mTor activity, overlapping with PI3K/AKT mutant clones in the fin mesenchyme prior to normal cartilage condensation (Fig. S1). Therefore, we hypothesize that clones with overctivated PI3K/AKT signaling induce premature condensation through both increasing cell density and upregulation of SOX9 levels.

The high proliferation rate of PI3K/AKT mutant mesenchymal clones in the larval fin suggests that the mutation can occur relatively late during embryogenesis, while still resulting in a sufficiently high number of mutant cells prior to cartilage formation. Consistent with this hypothesis, we did not find large mesenchymal clones in adjacent trunk regions in the majority of fish, suggesting that the expansion and overproliferation of PI3K/AKT mutant clones occurred only after recruitment of progenitors into the fin.

Heterochrony is a pervasive developmental mechanism in evolution, underlying shifts in morphology and physiology (de Beer, 1930; Gould, 1977). Through changes in developmental timing, broad changes in morphology can arise while remaining integrated and patterned. How the differential timing of growth underlying heterochrony is regulated is generally unknown but, most likely, it is multifaceted. Our work shows a particular case in which qualitative shifts in size are modulated within a distinct window during the pre-chondrogenic phase of skeletal anlagen formation.

We modeled localized, static overgrowth of long bones independently of other clinical presentations of PROS and Proteus syndrome. However, patients with PIK3CA- or AKT1-driven macrodactyly or enlarged skeletal bones can present with either static or progressive overgrowth (Cerrato et al., 2013; Keppler-Noreuil et al., 2014, 2015). Therefore, heterochrony is only a component of the developmental modulation of oncogenic clone behavior in these disorders. It remains to be shown whether the differential growth dynamics of static or progressive growth is due to modulating factors, or independent mechanisms, such as PI3K/AKT overactivation in a different cell type.

## MATERIALS AND METHODS

### Zebrafish husbandry, strains and staging

The study was conducted with ethical approval from the Institutional Animal Care and Use Committee of Boston Children's Hospital. Zebrafish (*Danio rerio*) were maintained under standard conditions (Westerfield, 2000). A description of the husbandry and environmental conditions at Boston Children's Hospital is available at protocols.io. All experiments were performed in the *casper* mutant background (White et al., 2008) to ensure optical accessibility of postembryonic stages. The following transgenic zebrafish strains were used: *Tg(sox10:DsRed)^el10Tg^* (Das and Crump, 2012) and *Tg(Ola.Sp7:mCherry)^zf131^* (DeLaurier et al., 2010). Standard length (SL) was used for staging of larvae and juvenile fish according to Parichy et al. (2009). In all experiments, we used size- and age-matched fish, and both sexes were included. Fish that did not match size criteria were excluded from experiments. The investigator was not blinded to group allocation during the experiments and analysis.

### Generation of mosaic zebrafish

The following plasmids were constructed for injection: *pmTol2-ubi:eGFP* (control construct), *pmTol2-ubi:akt^1E18K^-ubi:eGPF*, *pmTol2-ubi:myr-p110-ubi:eGPF* and *pmTol2-hsp70:akt^1E18K^-ubi:eGPF*. Plasmid *pminiTol2* (Addgene #31829) was used as backbone vector and transgene cassettes *ubi:eGFP*, *ubi:akt1^E18K^*, *ubi:myr-p110* and *hsp70:akt1^E18K^* were inserted into the multiple cloning site. Transgene cassettes were generated by inserting *eGFP* from *pME-eGFP* (Tol2kit #383), murine *myr-p110* from *Myr-(iSH2-p85)-p110alpha-Myc* (Addgene #1410) or zebrafish *akt1^E18K^* together with the SV40 late polyadenylation signal (SV40pA) downstream of the *ubi or hsp70* promoter into *pENTR5'_ubi* (Addgene #27320) or *p5E-hsp70I* (Tol2kit #222), respectively. The full-length coding sequence of zebrafish *akt1* was amplified from 24 h post fertilization cDNA and the E18K mutation was introduced using QuickChange Site-Directed mutagenesis (Agilent). To generate mosaic fish, plasmid DNA (5 ng/μl) and Tol2 transposase mRNA (4 ng/μl) (synthesized from *pT3TS-Tol2* Addgene #31831) were injected into one-cell stage embryos. Optimal plasmid DNA and *Tol2* mRNA concentrations were determined by titration experiments. We started with a plasmid DNA concentration of 20 ng/μl and a *Tol2* mRNA concentration of 20 ng/μl, which resulted in high numbers of transgene-expressing clones, as well as in high lethality in fish injected with *pmTol2-ubi:akt1*^*E18K*^*-ubi:eGFP* or *pmTol2-ubi:myr-p110-ubi:eGFP*. Concentrations of plasmid DNA and *Tol2* mRNA were gradually lowered until a 80-90% survival rate into adulthood was achieved.

### Skeletal staining

Adult fish were fixed for 1-2 days in 3.7% formaldehyde in phosphate-buffered saline (PBS). After fixation, fish were briefly rinsed with PBS and gradually transferred into 95% ethanol. Subsequently, fish were washed with acetone for 6 h to remove fat. Fish were then briefly rinsed with 95% ethyl alcohol and gradually transferred into PBS. Fish were equilibrated in 0.5% potassium hydroxide (KOH) and stained for 24 h in 0.1% Alizarin Red S (Sigma) in 0.5% KOH. After staining, fish were rinsed with 0.5% KOH followed by PBS. Next, fish were macerated using 3% trypsin (Fisher Scientific) in 30% saturated sodium borate at 37°C for 2-4 h. Digestion was stopped by briefly rinsing fish with PBS. Subsequently, fish were further cleared in 2% KOH for 24 h and gradually transferred to 75% glycerol in 0.5% KOH for storage and imaging. If not otherwise stated, all steps were carried out at room temperature under gentle rocking.

### Imaging

Stained fish skeletons were imaged using a Nikon SMZ18 stereomicroscope and NIS-Elements software. Adult fish were anesthetized with Tricaine mesylate (MS-222; 0.04%, Western Chemical Inc.) and photographed using a Canon EOS digital camera. Imaging of live and stained larval fish were performed using a Zeiss LSM 800 confocal microscope and ZEN Blue software or a Nikon SMZ18 stereomicroscope and NIS-Elements software for low magnification images of whole larvae. Larvae were anesthetized with MS-222 and embedded in 0.5% low-melting agarose for imaging. Confocal *z*-stacks were processed using ZEN blue software.

### Anti-pS6 immunostaining

Larvae were fixed at 4°C in 4% paraformaldehyde (PFA) in PBS overnight, rinsed 5× for 20 min in PBTx (PBS, 0.3% Triton X-100) and blocked in 5% goat serum. Subsequently, larvae were incubated with rabbit polyclonal antibody against S6 ribosomal protein (pS6) phosphorylated at serine residues 240 and 244 [Phospho-S6 Ribosomal Protein (Ser240/244), Cell Signaling, #2215] diluted 1:200 in 5% goat serum for 5 h at room temperature, rinsed 5× for 20 min in PBT (PBS, 0.1% Tween-20) and incubated overnight at 4°C with goat anti-rabbit antibody conjugated to Alexa Fluor 568 (Invitrogen, #A-11011) diluted 1:500 in PBT. Larvae were rinsed 5× in PBT and mounted for imaging in 0.5% low-melting agarose in PBS.

### EdU labeling and quantification

For EdU labeling, larvae were incubated in 2.5 mg/ml EdU (VWR) in PBS for 30 min. Larvae were fixed in 4% PFA in PBS overnight at 4°C and rinsed 5× for 20 min in PBTx. Larvae were equilibrated in 100 mM Tris-HCl pH 8 for 2 min and EdU was detected by using a copper-catalyzed azide-alkyne click reaction (25 µM Alexa Fluor 647 Azide (Invitrogen, A10277), 100 mM Tris, 1 mM CuSO4, 100 mM ascorbic acid, pH 8) with 30 min incubation time. Subsequently, larvae were rinsed 5× for 20 min in PBTx. The GFP signal was recovered by using an anti-GFP antibody. Immunostaining was performed as described for anti-pS6, with the following antibodies and dilutions: rabbit anti-GFP antibody (GeneTex, GTX113617; 1:200), goat anti-rabbit antibody conjugated to Alexa Fluor 488 (Invitrogen, A-11070; 1:500). For quantification of EdU^+^ caudal fin mesenchyme, cells within 300 µm anterior of the notochord tip were counted.

### Heat-shock treatment

Heat-shock treatment was performed in 100×25 mm Petri dishes. Heat shock was given at 38°C every 24 h for 2 h.

## Supplementary Material

10.1242/dmm.049793_sup1 Supplementary information
