# Supplementary material for: Localized heterochrony integrates overgrowth potential of oncogenic clones
Source: Dis Model Mech. 2023 Feb 8;16(2):dmm049793. doi: 10.1242/dmm.049793 (PMC9932785; doi:10.1242/dmm.049793)
Supplement: Supplementary information [file dmm-16-049793-s1.pdf]

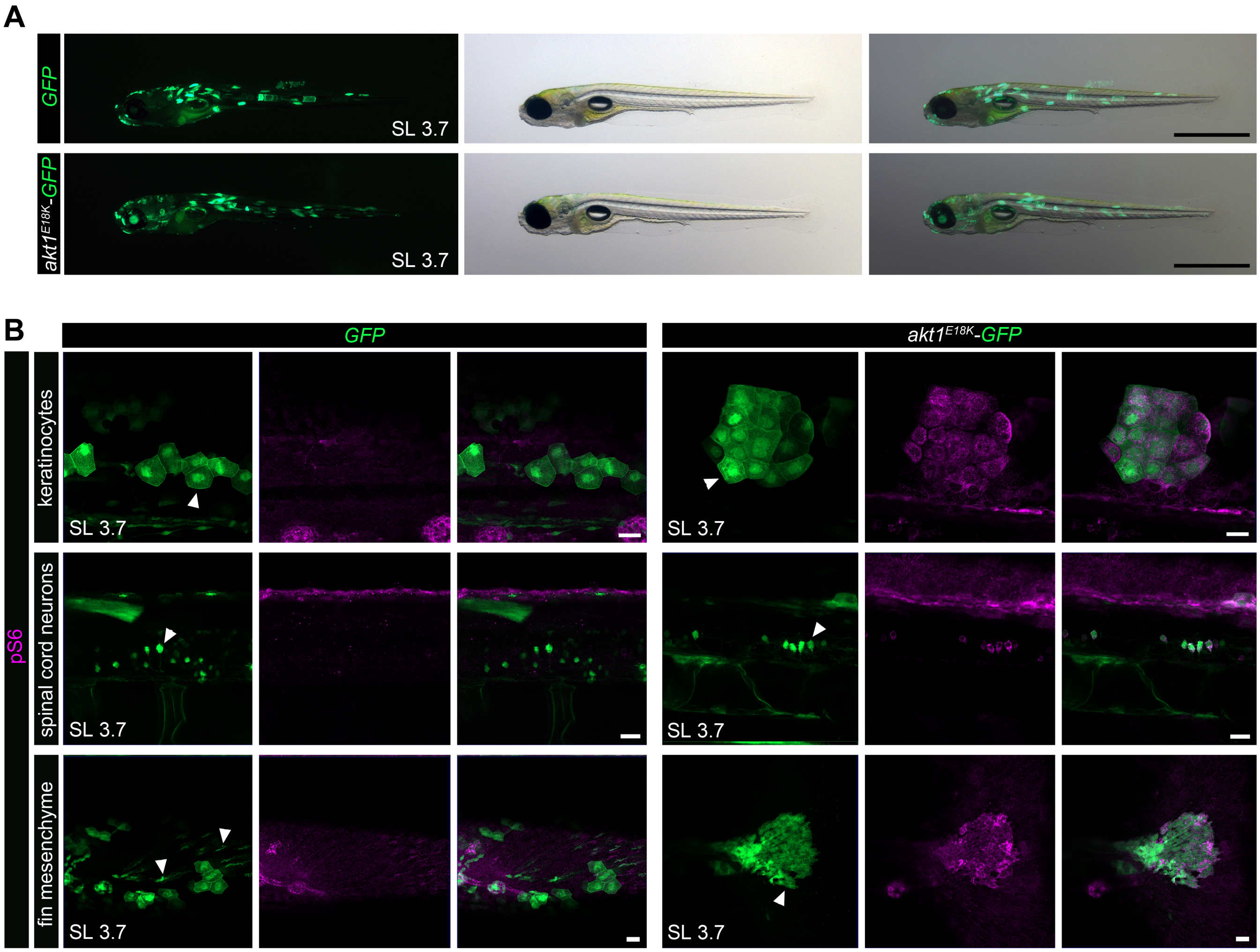

Fig. S1. The mTOR pathway is overactivated in *akt1^{E18K}-GFP mutant clones.*

(**A**) Examples of injected mosaic larvae with clones in multiple tissues. (**B**) Levels of pS6 staining are higher in *akt1^{E18K}-GFP* cells compared to cells expressing only *GFP.* Representative examples of pS6 immunofluorescence staining in keratinocytes, spinal cord neurons and mesenchymal cells in the larval caudal fin. Arrowheads mark cells of interest. SL, standard length in mm. Scale bars (**A**) 1 mm, (**B**) 20 µm.

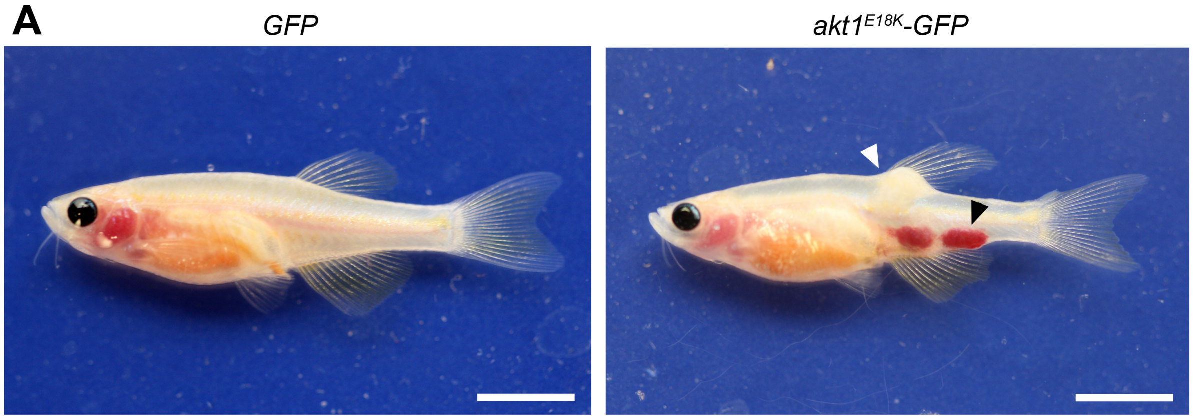

B

n=	*GFP*	*akt1^E18K^-GFP*	*myr-p110-GFP*
**skin**	**0**	**37**	**54**
**adipose tissue**	**0**	**49**	**13**
**muscle**	**0**	**9**	**3**
**vascular**	**0**	**9**	**1**
**analyzed fish**	**250**	**203**	**132**

Fig. S2. Zebrafish models for PROS and Proteus syndrome mimic patient phenotypes.
(**A**) Representative examples of adult *GFP* and *akt1*^{E18K}*-GFP* mosaic fish. Localized overgrowth of adipose tissue (white arrowhead) and vasculature (black arrowhead) is visible in *akt1*^{E18K}*-GFP* fish. (**B**) Quantification of externally visible overgrowth phenotypes in 3-6 months old fish. Scale bars, 0.5 cm.

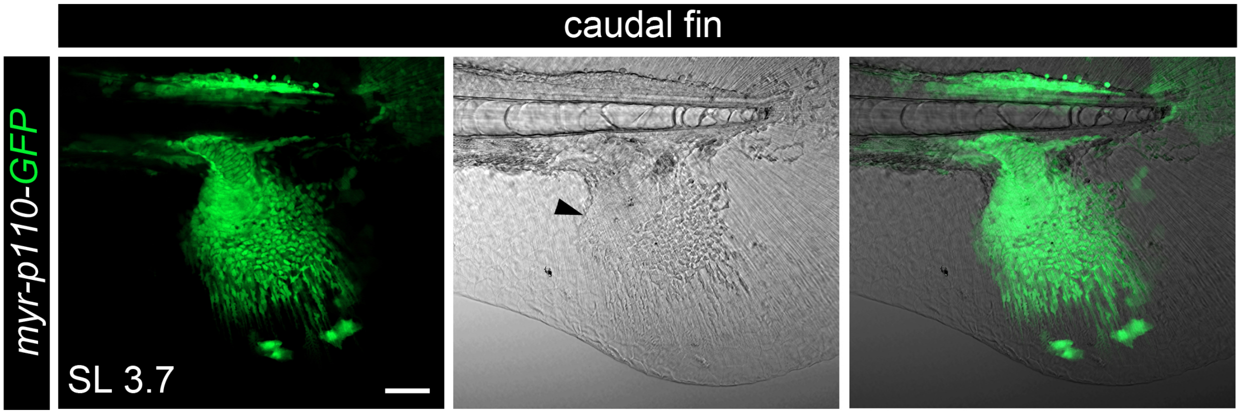

Fig. S3. PIK3CA mutant mesenchymal clones form premature pre-chondrogenic condensations.

In vivo imaging of *myr-p110-GFP* mesenchymal clones at SL 3.7 mm reveals clonal expansion and premature pre-chondrogenic condensation in the caudal fin. Arrowhead marks the edge of condensation. SL, standard length in mm. Scale bars, 50 µm.

Disease Models & Mechanisms • Supplementary information
